# Self-Assembly of Amyloid Fibrils That Display Active Enzymes

**DOI:** 10.1002/cctc.201402125

**Published:** 2014-06-04

**Authors:** Xiao-Ming Zhou, Aiman Entwistle, Hong Zhang, Antony P Jackson, Thomas O Mason, Ulyana Shimanovich, Tuomas P J Knowles, Andrew T Smith, Elizabeth B Sawyer, Sarah Perrett

**Affiliations:** [a] National Laboratory of Biomacromolecules, Institute of Biophysics, Chinese Academy of Sciences15 Datun Road, Chaoyang District, Beijing 100101 (China); [b] Department of Biochemistry, University of CambridgeTennis Court Road, Cambridge CB2 1QW (UK); [c] Department of Chemistry, University of CambridgeLensfield Road, Cambridge CB2 1EW (UK); [d] School of Applied Sciences, RMIT UniversityLa Trobe Street, Melbourne, Victoria 3000 (Australia); [e] University of the Chinese Academy of Sciences19 A Yuquanlu, Shijingshan District, Beijing 100049 (China)

**Keywords:** enzyme catalysis, immobilization, kinetics, microreactors, nanostructures

## Abstract

Enzyme immobilization is an important strategy to enhance the stability and recoverability of enzymes and to facilitate the separation of enzymes from reaction products. However, enzyme purification followed by separate chemical steps to allow immobilization on a solid support reduces the efficiency and yield of the active enzyme. Here we describe polypeptide constructs that self-assemble spontaneously into nanofibrils with fused active enzyme subunits displayed on the amyloid fibril surface. We measured the steady-state kinetic parameters for the appended enzymes in situ within fibrils and compare these with the identical protein constructs in solution. Finally, we demonstrated that the fibrils can be recycled and reused in functional assays both in conventional batch processes and in a continuous-flow microreactor.

## Introduction

Amyloid fibrils are stable, highly ordered polypeptide aggregates, the assembly of which can be controlled in vitro by altering conditions such as temperature, degree of agitation, and the presence of cosolvents. Several diseases are associated with the accumulation of amyloid fibrils,^1^ yet examples of amyloid fibrils that occur naturally with positive biological functions have also been identified recently, which include the curli proteins of *Escherichia coli* and Ure2 of *Saccharomyces cerevisiae*.^2^ Fibril morphology and structure are affected by the assembly conditions and the presence, position, and conformation of molecules appended to the amyloidogenic domain.^3^ The extraordinary stability and tunable assembly of amyloid fibrils make them attractive targets as nanomaterials;^4^ indeed, the functionalization of amyloid fibrils by metal binding and cytochrome display has already been explored^5^ and significant signal enhancements have been demonstrated if amyloid fibrils are employed in immuno- or biosensor assays.^6^

Ure2 is a regulator of nitrogen metabolism in *S. cerevisiae* through its conformation-dependent interactions with the transcription factor Gln3. The fibrillar form of Ure2 propagates in a manner analogous to mammalian prions,2b which makes it a useful tool to enhance the understanding of prions in general. Although no high-resolution structural data have yet been obtained, several recent structural studies on Ure2 fibrils have proposed the following model: the N-terminal prion domain (residues 1–93), which is rich in Asn and Gln residues and is necessary and sufficient for fibril formation in vitro and prion propagation in vivo^7^ forms a cross-β fibril core, around which the C-terminal globular domains are displayed.^8^ The C-terminal domain (CTD), a structural homologue of glutathione transferase enzymes,^9^ forms a homodimer and shows glutathione peroxidase and glutaredoxin activities in both the soluble and fibrillar forms of the protein.^10^

The central core of the Ure2 fibril is formed from the first 80 residues.8b Residues 81–100 provide flexibility between the prion domain and the CTD; this is important for the amyloid formation of Ure2^11^ and also offers the possibility of replacing the CTD with other proteins.8a Although the appendage of globular proteins to some amyloidogenic peptides requires that the globular protein first unfolds for fibrils to form,^12^ the globular C domain of Ure2 is accommodated in its native state within the fibrils.8a, 10

The value and use of commercial and industrial enzymes can be enhanced by immobilization. A key advantage of this process is the introduction of a phase separation between the enzyme and the reaction mixture, which facilitates the recovery and reuse of the enzyme and purification of the product. Immobilization has relied traditionally on four strategies: adsorption, covalent attachment of enzymes to carriers, entrapment, and chemical cross-linking.^13^ However, each of these methods may reduce the proportion of conformationally active enzyme, reduce flexibility, or limit the access of the substrate to the enzyme. Although some nanostructures, such as nanoparticles, nanofibers, and mesoporous silica, have been tested as immobilization materials to improve the surface-area-to-volume ratio, limitations in terms of dispersion in solution, a substrate diffusion barrier, efficiency of enzyme immobilization, and ability to recycle still remain.^14^ Thus, there is a significant driving force for the development of alternative methods for enzyme immobilization.

Several recent studies indicate a strong interest in the possibility of using amyloid fibrils as a scaffold for the immobilization of proteins of biological or chemical interest.3b, 4–6 Chemical cross-linking has been used to attach an enzyme to an amyloid scaffold.4b However, the cross-linking approach is inevitably hard to control and leads to heterogeneity of the system and potential modification of the molecule of interest. In contrast, the genetic fusion approach applied here leads to highly efficient and homogenous self-assembly. Previous studies have used the prion domain of another yeast prion protein, Sup35, as the fibril-forming component of a fusion protein nanowire that comprises monomeric proteins that fold reliably when overexpressed.^6^ Likewise, an earlier study indicated the potential of the Ure2 prion domain to form fibrils containing appended enzymes, at least for proteins that are expressed and folded easily.8a In contrast, we have explored the possibility of using the Ure2 prion domain to display enzymes with specific folding requirements and diverse architectures and we have chosen alkaline phosphatase (AP) and horseradish peroxidase (HRP) as industrially important enzyme models. We exploit the homogeneity of the system to perform quantitative characterization of enzymatic parameters for both soluble and fibrillar forms of the genetically fused constructs. Finally, we demonstrate the practical application of the enzymatically active fibrils to immobilize and, thereby, recycle and reuse the enzymes in both traditional batch processes and as the basis for flow chemistry in microfluidic channels.

## Results and Discussion

### Chimeric AP and HRP form fibrils with the cross-β architecture characteristic of amyloid

The prion domain of Ure2 was attached to the N terminus of AP or HRP, both of which have widespread applications in clinical and immunodiagnostic use and can be assessed for activity using simple colorimetric, fluorescent, or luminescent assays even at low (single molecule) concentrations, which raises the possibility of using our chimeras to probe the assembly and subunit structure of Ure2. AP assembles as a mirror symmetric dimer (unlike the wild-type (WT) Ure2 dimer, which has axial symmetry) and catalyzes the hydrolysis of mono- and diphosphoesters from a range of substrates. HRP is a monomeric hemoglycoprotein enzyme capable of the very rapid turnover of a range of synthetic substrates. It forms inclusion bodies if overexpressed in *E. coli* and requires refolding in vitro (which includes the formation of four disulfide bonds and the incorporation of one heme cofactor and two Ca^2+^ ions) to achieve its active conformation^15^ (see Supporting Information).

**Figure 1:**
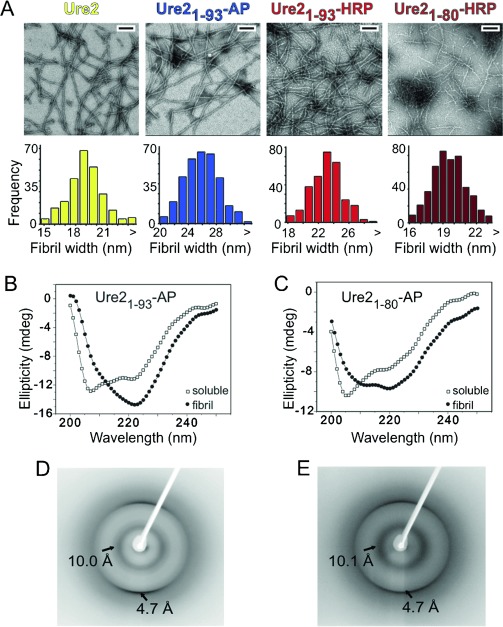
Chimeras between the Ure2 prion domain and AP or HRP enzymes form amyloid fibrils. A) Upper panel: negative-staining TEM of WT Ure2 and chimeric fibrillar aggregates. All scale bars 200 nm. Lower panel: widths of Ure2 and chimeric fibrils measured from TEM images. WT Ure2, Ure2_1−93_-AP, and Ure2_1−93_-HRP show median widths of 19, 26, and 23 nm (*n*>300), respectively, consistent with their relative molecular weights. Ure2_1−80_-HRP fibrils have a smaller diameter than HRP chimeric fibrils that contain the full-length prion domain of Ure2 (residues 1–93). CD spectra of native (open squares) and fibrillar (closed circles) B) Ure2_1−93_-AP and C) Ure2_1−80_-HRP. Fibril assembly of chimeric proteins is associated with an increase in β-sheet content. X-ray fiber diffraction patterns of D) Ure2_1−93_-AP fibrils and E) Ure2_1−80_-HRP fibrils, which show anisotropic reflections at 4.7 and 10 Å.

All the chimeric proteins formed fibrillar aggregates, which were characterized by negative-staining TEM, circular dichroism (CD) spectroscopy, and X-ray fiber diffraction (Figure 1). The fibril morphologies were similar to that of WT Ure2 and their widths were consistent with the relative monomeric molecular weights of the soluble proteins (WT Ure2 CTD, 29.8 kDa; AP, 49.4 kDa; HRP, 34.0 kDa). CD spectroscopy revealed a significant increase in the proportion of β-sheet secondary structure after fibril formation, consistent with the assembly of proteins into cross-β amyloid fibrils (Figure 1 B and C), and X-ray fiber diffraction of dry stalks gave rise to anisotropic reflections at 4.7 and 10 Å for Ure2_1−93_-AP and Ure2_1−80_-HRP fibrils, respectively, characteristic of the presence of a cross-β fibril core^16^ (Figure 1 D and E) similar to that of WT Ure2.8d, 17

### AP and HRP chimeric fibrils show enzymatic activity: Insight into the effects of enzyme immobilization from steady-state kinetic parameters

Similar to WT Ure2,^10^ our chimeras retained their enzyme activities after fibril formation. Activities were measured for both soluble and fibrillar samples at a range of substrate concentrations, and the steady-state enzyme kinetics data for the chimeras were compared to those of WT AP and WT HRP. The substrates used were *p*-nitrophenyl phosphate (pNPP) and 2,2′-azinobis-(3-ethylbenzthioazoline-6-sulfonic acid) diammonium salt (ABTS) for AP and HRP, respectively. The kinetic parameters obtained are shown in Table 1. Attachment of the Ure2 prion domain to the N terminus of AP appeared to lower the affinity of the enzyme for the substrate after the formation of amyloid fibrils, as suggested by an increase in the apparent Michaelis constant (*K*_M_), whereas the soluble chimera showed a more similar *K*_M_ value to that of WT AP. Soluble and fibrillar forms of Ure2_1−93_-AP showed similar catalytic activities, around 10-fold lower than that of WT AP, as indicated by the turnover number at saturation, *k*_cat_. This suggests that the observed reduction in catalytic activity is a result of the addition of the fusion itself, which may partially compromise the folded structure of AP. However, within the fibrils, the native AP molecules are displayed in a manner that is readily accessible to the substrate and catalyzes the phosphatase reaction with similar efficiency to the soluble form of the same enzyme construct.

**Table 1:** Kinetic parameters *k*_cat_ and *K*_M_ derived from the analysis of the rate of reaction of various AP and HRP constructs under steady-state conditions using pNPP and ABTS as substrates, respectively.

Enzyme	Construct	*k*_cat_ [s^−1^][a]	*K*_M_ [μm][a]	*k*_cat_/*K*_M_ [μm^−1^ s^−1^][a]
AP	WT AP	45.3±1.4	23±1	1.97
	Ure2_1−93_-AP	3.3±0.1	35±5	0.09
	Ure2_1−93_-AP (fibril)	4.0±0.3	94±24	0.04
HRP	WT HRP	592±18	412±22	1.44
	Ure2_1−80_-HRP	219±13	258±25	0.85
	Ure2_1−93_-HRP	155±17	197±39	0.79
	Ure2_1−80_-HRP (fibril)	72±3	604±51	0.12
	Ure2_1−93_-HRP (fibril)	31±2	410±36	0.08

[a] [a] The errors shown are the standard error of the fit of the data shown in Figure S3 in the Supporting Information.

The full-length prion domain (residues 1–93) was fused to the N terminus of HRP, but the activity of the resulting fibrils was low. The shortening of the prion domain (using only residues 1–80, which has previously been shown to allow fibril formation of the prion domain8b) led to an approximate doubling of the catalytic activity for the fibrillar form. As a result of the nature of the peroxidase reaction cycle (a ping pong mechanism dominated by compulsory second-order steps^18^), the apparent *K*_M_ cannot be equated directly with substrate affinity. The ratio of *k*_cat_/*K*_M_ is, however, a relative estimate of the slowest second-order step *k*_3_ (the rate for substrate association, which corresponds to the reduction of compound II to resting enzyme^19^) in the catalytic cycle.^18^ This parameter shows a dramatic decrease for the fibrillar form compared with the soluble form. The most probable explanation for this is reduced enzyme activity because of the steric hindrance of substrate access to the aromatic donor binding site at the reactive heme edge (centered on Phe 179 in WT HRP^18^). The more marked effect on catalytic activity upon fibril formation for HRP compared to AP is consistent with the very high turnover number of HRP, which means that the rate of catalysis is essentially diffusion controlled.

### Chimeric fibrils of AP and HRP display a similar fibril core structure to WT Ure2

**Figure 2:**
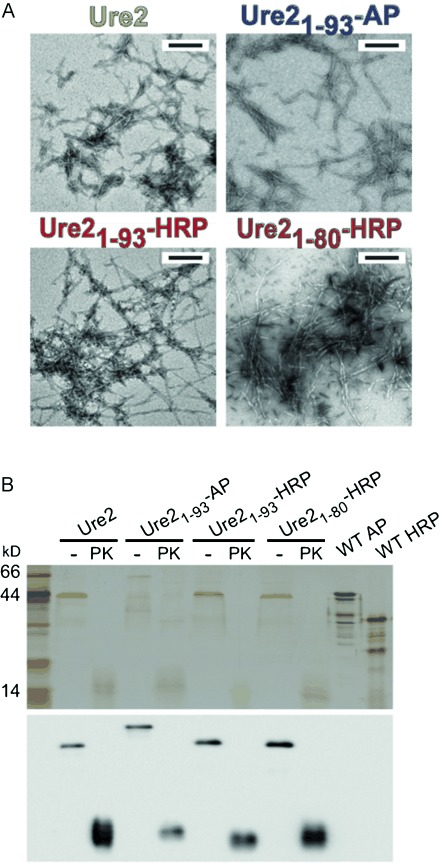
Chimeric fibrils of AP and HRP display a similar fibril core structure as WT Ure2. A) Negative-staining TEM images of WT Ure2 and chimeric protein fibrils after proteinase K (PK) digestion. All scale bars 200 nm. B) Anti-Ure2 Western blot (lower panel) of insoluble pellets of WT Ure2 and chimeric protein fibrils with or without PK digestion, centrifuged and washed with buffer three times, and dissolved in 8 m urea prior to SDS-PAGE (upper panel). The similarity in widths and morphologies of the fibril cores, and immunospecificity to Ure2 antibody of digested chimeric fibrils, suggests that the chimeric fibrils and WT Ure2 share a common fibril core.

The fibril core can be identified by its resistance to proteases. We used proteinase K digestion and Western blotting to assess the composition of the fibril cores of our chimeric protein fibrils. All fibrils were treated for at least 24 h with 0.5 mg mL^−1^ proteinase K, a broad-spectrum serine protease that cleaves next to aliphatic and aromatic residues, and the fibril cores that remained were analyzed by TEM. Similar core widths (6–8 nm) were observed for all samples (Figure 2 A), and washed pellets that contained only the fibril cores did not show any enzymatic activity against the test substrates.

The composition of the fibril cores was also tested by Western blot using an anti-Ure2 primary antibody. After proteinase K digestion, the fibril cores were washed with buffer and dissolved in 8 m urea with 2 % sodium dodecyl sulfate (SDS) for polyacrylamide gel electrophoresis (PAGE). Protein bands that correspond to the fibril cores do not stain efficiently with Coomassie Blue, but silver staining revealed smeared bands around 13 kDa in size, which correspond to the dissolved fibril core. These bands showed immunospecificity to the anti-Ure2 antibody, whereas degraded WT AP and HRP (negative controls) were not recognized by the antibody (Figure 2 B).

**Figure 3:**
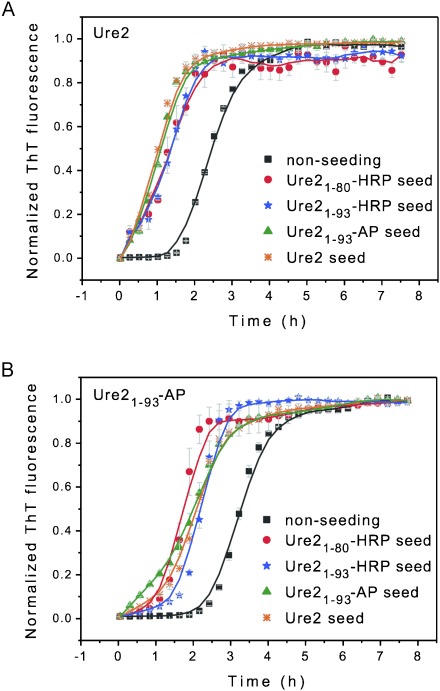
Cross-seeding of chimeric fibrils and WT Ure2 monitored by ThT fluorescence. A) WT Ure2 and B) Ure2_1−93_-AP each seeded with WT Ure2 or chimeric fibril seeds as indicated. The lag phases of WT Ure2 and Ure2_1−93_-AP fibril formation were reduced by seeding. (Errors shown are the standard error of the mean of three measurements. See Figure S1 in the Supporting Information.)

Further evidence that the chimeric proteins share the same fibril core as WT Ure2 comes from cross-seeding assays. The fibril formation of Ure2 and Ure2_1−93_-AP was seeded with WT Ure2 and chimeric protein fibril seeds and monitored by thioflavin T (ThT) fluorescence. A decrease in the lag phase of WT Ure2 and Ure2_1−93_-AP fibril formation was observed after seeding (Figure 3 A and B).

The self-assembly of Ure2_1−93_-HRP and Ure2_1−80_-HRP was monitored by TEM because fibril formation did not give rise to any change in ThT fluorescence, perhaps because of quenching by HRP-bound heme. Large fibrils were observed after seeding, whereas no fibrils were observed in the nonseeded samples (Figure S1 A and B in the Supporting Information). All fibril seeds appeared as short rods in the TEM images, and no change was observed after 3 h incubation under the conditions of the seeding assay in the absence of soluble protein (Figure S1 C in the Supporting Information). WT Ure2 and Ure2 chimeras were able to cross-seed each other’s growth, which suggests that the stacking within these fibrils (i.e., the structure of the fibril core) is similar. This is consistent with a model in which the fibril cores are formed of the prion domain of Ure2, and AP/HRP is displayed on the surface, which has been suggested previously for fibrils of WT Ure2.^8^

### Chimeric fibrils can be recycled in functional assays in both batch processes and in a continuous-flow microreactor

One of the primary goals of enzyme immobilization is the ability to recover and reuse enzymes. This is significant economically but also facilitates subsequent purification of the product by the full removal of one component of the reaction mixture in bulk. Our chimeric fibrils could, for instance, be reacted with substrate repeatedly, recovered by centrifugation, washed, and then recycled with minimal loss of yield or activity after each round of reaction and recycling (Figure S2 in the Supporting Information).

**Figure 4:**
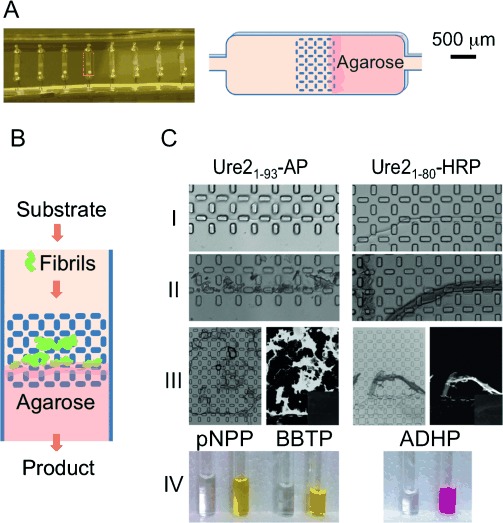
Application of chimeric fibrils in a continuous-flow microreactor. Fibrils were trapped in a microcolumn, which allowed the substrate to be introduced through continuous flow and the reaction product was collected at the outlet. A) Diagram of the structure of the microcolumn (right panel) indicated in the photograph by a red box (left panel). The microcolumn was fabricated using soft lithography techniques into polydimethylsiloxane.^20^ B) Schematic of flow chemistry. C) Bright-field images of agarose interface used to trap fibrils (Panel I), and stacking of the fibrils on the agarose interface (Panel II). Panel III: bright-field image (left) and ThT fluorescence image (right) of trapped fibrils. Weak intrinsic fluorescence of fibrils (right, inset). Panel IV: colorless substrates were loaded into the column and colored products were collected. See Experimental Section for further details.

In addition to enzyme recovery through batch processes such as centrifugation, the availability of the enzymatically functionalized nanoscaffold architecture developed in this study also opens up the possibility of using flow chemistry to perform reactions under continuous-flow conditions in which the separation of the active enzymes from the reaction products occurs in a continuous manner. We demonstrated this idea by packing chimeric fibrils into a microcolumn and found that the substrate could be readily pumped in and the product could be collected in the flow-through (Figure 4). The fibrils could be washed repeatedly and reused with the same or different substrates. No cross-reactivity between substrates was found after washing with buffer. This finding confirms that the fibrils are stable in dilute solution and can be efficiently recycled and reused. Furthermore, they can be readily applied in microscale detection and high-throughput measurements.

To further confirm whether the level of activity retained in our fibril-immobilized enzymes has any potential commercial or industrial application, we tested the possibility of using Ure2_1−93_-AP fibrils in place of shrimp alkaline phosphatase (SAP). SAP is used routinely in molecular biology to remove 5′ phosphate groups from DNA to prevent self-ligation. After reaction, it is necessary to remove or inactivate the SAP so that the target DNA insert is not also dephosphorylated. This is normally achieved by irreversible heat inactivation of the enzyme by incubation at 70 °C for 30 min. To test whether Ure2_1−93_-AP fibrils are as effective as SAP at dephosphorylating DNA, a blue/white selection assay was used. Top10 *E. coli* were transformed with DNA that had been treated with buffer, SAP, or Ure2_1−93_-AP fibrils before ligation, and the proportion of blue/white colonies was determined. Blue colonies indicate the religation of the vector without gene insertion, whereas white colonies indicate successful gene insertion.

**Figure 5:**
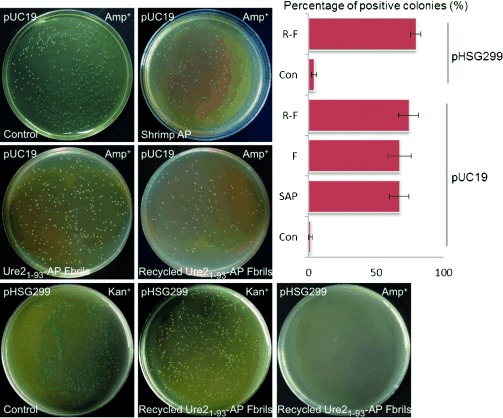
Application of AP chimeric fibrils in a molecular biology assay. Plasmids (as indicated) treated with buffer (Con, control), SAP, Ure2_1−93_-AP fibrils (F), or recycled Ure2_1−93_-AP fibrils (R-F) were transformed into Top10 cells for blue/white selection. White colonies on the plates indicate successful phosphatase treatment and gene insertion. The proportion of white colonies after treatment is represented in the graph. Ure2_1−93_-AP fibrils are at least as effective as SAP but more convenient to use and can be recycled. Furthermore, for the pHSG299 plasmid treated with recycled Ure2_1−93_-AP fibrils, the absence of any colonies growing on the Amp^+^ plate indicates that plasmid contamination of reused Ure2_1−93_-AP fibrils is absent. See Experimental Section for further details.

The immobilized AP was recycled and re-reacted with the same linear plasmid (pUC19) to test its recyclability. Meanwhile, another plasmid (pHSG299) with different antibiotic resistance was used to check for plasmid contamination from the previous reaction when Ure2_1−93_-AP fibrils are reused. The results of the screening assay are shown in Figure 5. Not only do Ure2_1−93_-AP fibrils catalyze the phosphatase reaction as efficiently as commercial SAP but they can also be recovered and recycled with the same proportion of positive colonies and without plasmid contamination from previous reactions. Furthermore, the ability to separate Ure2_1−93_-AP fibrils easily from the reaction mixture, and the lack of requirement for heat denaturation, means that immobilized AP is more convenient to use than SAP. Thus, Ure2_1−93_-AP fibrils offer a sustainable and cost-effective alternative to the use of SAP in molecular biology.

## Conclusions

In our system, the prion domain of Ure2 provides a naturally stable linker between the fibril core and enzymatic domain and allows the immobilization of the enzymes with high efficiency and homogeneity. The tunable self-assembly of our system, the ability to separate enzymes from substrate/product by centrifugation or trapping within a column, and the excellent recyclability of our enzymatic fibrils satisfy the requirements for efficient enzyme immobilization. The enzymes we have chosen to append to the Ure2 prion domain demonstrate the capacity of this domain to support a variety of molecular sizes and architectures. Alkaline phosphatase is nearly twice the size of the Ure2 C-terminal domain and the arrangements of the dimers are completely unalike. Horseradish peroxidase (HRP) is a difficult protein to express in its native state from *E. coli*, and refolding from inclusion bodies is required. As a result of its varied and widespread commercial use, there is interest in the production of a recyclable form of HRP. Finally, we have demonstrated that the efficiency, stability, and homogeneity of our self-assembly system allows the direct comparison of enzyme kinetic parameters for the soluble and fibrillar forms of the same protein construct, which provides insight into the effects of immobilization and thus strategies for further optimization and development. Thus, the prion domain of Ure2 appears to be an ideal scaffold for the immobilization and display of active enzymes on amyloid fibrils as well as a new vehicle to understand fibril assembly.

## Experimental Section

### Materials

Unless otherwise stated, reagents were purchased from Sigma (Sigma–Aldrich co. LLC), and solutions were made using water purified to a resistance of 18.2 MΩ (Millipore). Protein concentrations were determined by absorbance at 280 nm using extinction coefficients of 48 220 (WT Ure2), 33 140 (AP constructs), or 13 050 m^−1^ cm^−1^ (HRP constructs; the prion domain of Ure2 contains no Tyr, Trp, or Cys residues), or by bicinchoninic acid (BCA) assay (Pierce Biotechnology Inc.) following the addition of 2 % SDS and incubation at RT for at least 1 h to dissolve fibrils.

### Protein expression and purification

Standard molecular cloning techniques were used to fuse together genes that encode the prion domain of Ure2 and AP (the *E. coli* K-12 gene was kindly provided by Prof. Qun Wei, Beijing Normal University, China) or HRP (synthetic HRP isoenzyme C (HRP-C)^21^). WT Ure2 and Ure2_1−93_-AP were cloned into a mini-pRSETa expression vector (kindly provided by Dr. Mark Bycroft, MRC LMB) and expressed with an N-terminal 6x-His tag. WT HRP and all HRP chimeras were cloned into pET24a expression vectors for expression without His tags.

WT Ure2, WT AP and Ure2_1−93_-AP were expressed in C41 *E. coli* in 2×YT media. The cells were grown at 37 °C to an optical density 600 nm of 0.6, induced with 0.4 mm isopropyl β-D-1-thiogalactopyranoside (IPTG) and grown at 16 °C for 20 h. The cells were harvested by centrifugation at 5000×g for 50 min and resuspended in buffer A (50 mm Tris-HCl, 150 mm NaCl, pH 8.0). Cells were then lysed by using a high-pressure cell press (JNBIO JN-3000 PLUS), and the insoluble material was removed by centrifugation at 30 000×g for 60 min. The soluble Ure2 and Ure2_1−93_-AP contained in the supernatants were applied to nickel-affinity chromatography resin (Chelating Sepharose Fast Flow, Amersham Biosciences) and purified according to manufacturer’s guidelines. Imidazole was removed from the purified proteins by buffer exchange into buffer A by using Amicon centrifugal concentrators (Millipore). Soluble WT AP was further purified by anion-exchange chromatography by using a Q Sepharose Fast Flow column (Amersham Biosciences). The purity of the proteins was estimated by SDS-PAGE to be >95 %.

WT HRP, Ure2_1−93_-HRP and Ure2_1−80_-HRP were expressed in BL21 *E. coli* at 16 °C in 2×YT media after induction with 0.2 mm IPTG. The cells were harvested by centrifugation at 5000×g for 50 min and resuspended in 50 mm Tris-HCl, 10 mm ethylenediaminetetraacetic acid (EDTA), and 1 mm 1,4-dithiothreitol (DTT), pH 8.0 buffer. Following pressure lysis, WT HRP, Ure2_1−93_-HRP, and Ure2_1−80_-HRP were purified and refolded from inclusion bodies using a modified version of the method described previously.^21^ In brief, the purified WT HRP, Ure2_1−93_-HRP, and Ure2_1−80_-HRP inclusion bodies were dissolved in 8 m Urea, 50 mm Tris-HCl, and 1 mm DTT, pH 8.0. The denatured proteins were refolded by stepwise dilution in 2 m urea that contained 50 mm Tris-HCl (pH 8.0), 8 mm CaCl_2_, and 0.7 mm glutathione disulfide (GSSG). First, the inclusion body mixture (100 mL) was added to an equal volume of refolding buffer, and heme was added to a final concentration of 10 μm. After 30 min, further folding buffer/inclusion body mixture (200 mL) was added into the original, and the heme concentration was adjusted back to 10 μm by further addition of stock solution. This was repeated until the final volume of the mixture reached 1 L over a period of approximately 17 h at 4 °C to a final protein concentration of 0.2 mg mL^−1^. Heme precipitate was removed by centrifugation, and the protein was concentrated by using an Amicon centrifugal concentrator (Millipore) to a final volume of 90–100 mL. The refolded WT HRP, Ure2_1−93_-HRP, and Ure2_1−80_-HRP were then dialyzed against buffer B (20 mm Na acetate, pH 4.3, that contained 1 mm CaCl_2_). Again, the purity of the proteins estimated by SDS-PAGE was >95 %. A UV/Vis spectrum of the protein preparations showed a five-coordinate high-spin spectrum typical of active peroxidase with Reinheitszahl (RZ; a measure of the hemin content by the absorbance ratio A403/A275) values of >2.7.

### Fibril formation

Ure2_1−93_-AP fibrils were formed by incubation at 4 °C for 2 days without shaking. WT Ure2, Ure2_1−93_-HRP, and Ure2_1−80_-HRP fibrils were formed by shaking at 220 rpm (Refrigerated Incubator Shaker, Innova 4230, New Brunswick Scientific) at 30 °C. The protein concentrations used for fibril formation were typically 50–70 μm. Fibril formation was monitored by ThT fluorescence as described previously^22^ and/or the resulting fibrils were characterized by negative-staining TEM. Conditions were chosen to allow the reproducible formation of fibrils of consistent lengths and diameters and without the loss of enzyme activity or the formation of amorphous aggregates.

### Electron microscopy

For negative-staining TEM, 6 μL drops of suspensions of fibrils or soluble proteins (protein concentration around 30 μm) were loaded onto glow-discharged carbon-coated grids for 1 min and blotted with filter paper to remove extra sample, then rinsed with deionized water (6 μL) and stained with 2 % uranyl acetate for 20 s. Micrographs were recorded by using a CM120-FEG (FEI) microscope operating at 100 kV.

### CD spectroscopy

CD spectra were obtained by using a Pistar-180 instrument at RT. All spectra were measured using a 1 mm pathlength quartz cuvette, 1 nm step size, and 2 nm slit width in buffer A for Ure2_1−93_-AP and buffer B for Ure2_1−80_-HRP, using protein concentrations of 6 and 3 μm, respectively.

### X-ray fiber diffraction

Chimeric fibrils were washed with deionized water three times to remove salts, then the washed fibrils were dried and aligned on the end of a glass capillary, as described previously.^23^ X-ray diffraction patterns of the dehydrated fibril stalks were collected by using a Rigaku R-AXIS IV++ image-plate system calibrated against high-density polyethylene (HDPE). The sample–detector distance was 250 mm and the exposure time was 30 s. The 2 D diffraction patterns were converted into tiff images by using fit-2D and integrated into 1 D scattering profiles by using Image J.^23^

### Western blot

Protein fibrils were washed with buffer three times, resuspended in buffer at concentrations of 40–70 μm, and incubated with 0.5 mg mL^−1^ proteinase K (Amresco) and 1 mm CaCl_2_ for more than 24 h at 37 °C. Insoluble material was collected by centrifugation at 10 000×g for 15 min at 4 °C, washed twice with buffer, dissolved in 8 m urea, and boiled in the presence of 2 % SDS for SDS-PAGE. Proteins in the gels were subjected to silver staining or transferred to polyvinylidene difluoride (PVDF) membrane for Western blot. A polyclonal anti-Ure2 antibody (made by Sino Biological Inc., China) was used to test the immunospecificity of the samples.

### Cross-seeding assay

To generate seeds, fibrils were diluted to 2–3 μm and sonicated (750 Watt Ultrasonic Processors, SONICS) at 30 % power for 1 min (1 s on and 1 s off) in Eppendorf tubes incubated on ice. The fibril seeds diluted with buffer A were used to seed the fibril formation of Ure2 and Ure2_1−93_-AP, and seeds diluted with buffer B were used to seed Ure2_1−93_-HRP and Ure2_1−80_-HRP fibril formation. Solutions of Ure2 and chimeric proteins (30–40 μm) contained 2–3 % seeds of Ure2 or Ure2_1−93_-AP, or 4 % seeds of Ure2_1−93_-HRP or Ure2_1−80_-HRP. For the Ure2-AP cross-seeding assays, Ure2 or Ure21–93-AP (150 μL; soluble protein or seeds) was loaded into 96-well plates and shaken at 30 °C. Fibril formation was monitored by ThT fluorescence (17 μm ThT) by using a plate-reader (SpectraMax M3 Multi-Mode Microplate Reader, Molecular Devices). For Ure2_1−93_-HRP and Ure2_1−80_-HRP, the seeding mixture was divided into 400 μL aliquots and incubated at 30 °C with 220 rpm shaking (Refrigerated Incubator Shaker, Innova 4230, New Brunswick Scientific). Fibril formation was monitored by negative-staining TEM (Ure2_1−93_-HRP and Ure2_1−80_-HRP fibrils do not exhibit ThT fluorescence, possibly because of quenching by the heme group of HRP).

### Enzyme activity assay and recycling of fibrils

Chimeric fibrils were washed twice with buffer and recovered by centrifugation. Ure2_1−93_-AP fibrils (600 μL, ∼30 μm) were allowed to react with pNPP (20 μL, 500 mm) for 10 min before recovery by centrifugation at 10 000×g for 10 min. The recovered fibrils were then washed with buffer A four times to ensure the complete removal of product, before beginning a new round of reaction with pNPP. The recycling was performed four times, and the mass and activity of the fibrils were measured at each round.

After reaction with ABTS, the Ure2_1−80_-HRP fibrils became purple and could not be washed back to their original color. These product-bound fibrils showed a significant loss of activity. Therefore, 3,3′,5,5′-tetramethylbenzidine (TMB), which did not show irreversible binding to fibrils, was used instead as the substrate. Ure2_1−80_-HRP fibrils (500 μL, ∼25 μm) were reacted with TMB (10 μL) for 10 min and recycled by centrifugation. The recycled Ure2_1−80_-HRP fibrils were then washed with buffer B once to remove products and reacted with TMB again. The recycling was performed four times. The Ure2_1−80_-HRP fibril mass and activity were monitored by BCA assay and ABTS, respectively, at each round of recycling.

To measure the steady-state kinetic parameters, WT AP was diluted to 0.005 μm and soluble and fibrillar Ure2_1−93_-AP were diluted to 0.05 μm and reacted with pNPP (New England BioLabs) in the range of 3.125–1000 μm. Dynamic light scattering measurements confirmed that fibrillar Ure2_1−93_-AP remained aggregated after dilution to 0.05 μm and incubation for 4 h. Product formation was monitored by absorbance at 405 nm for 10 min. For reactions with HRP-containing constructs, the ABTS was in the range 31.25–1500 μm. The protein concentrations used were as follows: WT HRP, soluble Ure2_1−80_-HRP, and soluble Ure2_1−93_-HRP 0.03 μm; fibrillar Ure2_1−80_-HRP and fibrillar Ure2_1−93_-HRP 0.06 μm. The peroxide concentration in all HRP assays was 2 mm. Product formation was monitored by absorbance at 405 nm for 5 min, and the initial rates of reaction were determined. The final specific activity of WT HRP was very similar to that reported previously.^15^

### Microcolumn assay

The microcolumn device was constructed using soft lithography techniques.^20^, ^24^ The device was fabricated in polydimethylsiloxane (PDMS; Dow Corning) using SU8 on silicon masters and plasma-bonded on a glass slide to seal the device. The size of the column was 5 mm × 1 mm × 0.02 mm to give a calculated volume of less than 0.1 μL. One end of the column was blocked with 1 % agarose introduced as a solution at 100 °C into half of the device and left to cool in situ to form a gel; the other end was left open. The Ure2_1−93_-AP and Ure2_1−80_-HRP fibrils were washed with buffer and diluted to around 15 μm, then the fibrils were injected and trapped into the column from the open side. The interface of the blocking agarose and stacking of the enzymatic fibrils were observed by light microscopy. ThT (20 μm) was pumped into the column for approximately 10 min, then excess ThT was washed out by pumping buffer through the column. The fluorescence of the trapped fibrils was monitored by fluorescence microscopy before and after loading with ThT. After first washing with buffer A, the colorless substrate pNPP (500 μm) was introduced into the Ure2_1−93_-AP microcolumn with a syringe pump, and the yellow product was collected from the outlet. After running pNPP, the Ure2_1−93_-AP microcolumn was washed with buffer A to remove any substrate or product. The fluorescent substrate 2′-(2-benzothiazoyl)-6′-hydroxybenzothiazole phosphate (BBTP, AttoPhos AP fluorescent Substrate System, Promega), was then pumped into the washed Ure2_1−93_-AP microcolumn, and the bright yellow product was collected. No cross reaction was detected between pNPP (or its product) and BBTP. The Ure2_1−80_-HRP microcolumn was first washed with buffer B and then the substrate 10-acetyl-3,7-dihydroxyphenoxazine (ADHP, QuantaRed Enhanced Chemifluorescent HRP Substrate, Thermo) was pumped in, and the bright pink product was collected.

### Blue/white selection assay

The gene fragment Ure2_1−93_-HRP fused with *Bam*H I sites at the two termini (B-UH-B) was amplified by polymerase chain reaction (PCR). The pUC19 plasmid (ThermoScientific) contains an ampicillin resistance gene and the *lac*Z gene, which encodes β-galactosidase, and contains a *Bam*H I site. The plasmid pHSG299 (Takara) is similar to pUC19 except that it has kanamycin resistance. The B-UH-B gene and pUC19 and pHSG299 plasmids were cut with *Bam*H I for 3 h at 30 °C and extracted from agarose gel. The linear pUC19 was treated with SAP (1000 units/mL, used at 1 μL enzyme per 10 μL of plasmid) or Ure2_1−93_-AP fibrils (around 50 μm, 1 μL fibrils per 10 μL of plasmid) for 1 h at 37 °C. Samples that contained SAP were incubated at 70 °C for 30 min to inactivate the SAP; samples that had been treated with Ure2_1−93_-AP fibrils instead of SAP were not subjected to this treatment, but the Ure2_1−93_-AP fibrils were removed by centrifugation at 12 000×g for 10 min and recycled. The recycled Ure2_1−93_-AP fibrils were washed twice with deionized water and used to treat the linear pUC19 and pHSG299 plasmids for 1 h at 37 °C, then the plasmids were separated from Ure2_1−93_-AP fibrils by centrifugation at 12 000×g for 10 min. All SAP- and Ure2_1−93_-AP fibril-treated plasmids were ligated to the B-UH-B gene (also cut with *Bam*H I) by T4 ligase for 12 h at 16 °C. As controls, linear pUC19 and pHSG299 were treated with buffer instead of SAP or Ure2_1−93_-AP before ligation with the gene fragments. All ligation products were transformed into Top10 cells and spread on 2×YT plates coated with IPTG and 5-bromo-4-chloro-3-indolyl-β-D-galactopyranoside (X-Gal). After approximately 14 h incubation at 37 °C, the plates were photographed and the blue (negative) colonies and white (positive) colonies were counted by using Image J software.
